# A rapid diagnostic test for human Visceral Leishmaniasis using novel *Leishmania* antigens in a Laser Direct-Write Lateral Flow Device

**DOI:** 10.1080/22221751.2019.1635430

**Published:** 2019-08-05

**Authors:** Maria Victoria Humbert, Lourena Emanuele Costa, Ioannis Katis, Fernanda Fonseca Ramos, Amanda Sanchéz Machado, Collin Sones, Eduardo Antonio Ferraz Coelho, Myron Christodoulides

**Affiliations:** a Neisseria Research Group, Molecular Microbiology, School of Clinical and Experimental Sciences, University of Southampton Faculty of Medicine, Southampton General Hospital, Southampton, England; b Programa de Pós-Graduação em Ciências da Saúde: Infectologia e Medicina Tropical, Faculdade de Medicina, Universidade Federal de Minas Gerais, Belo Horizonte, Brazil; c Optoelectronics Research Centre, University of Southampton, Southampton, England; d Departamento de Patologia Clínica, COLTEC, Universidade Federal de Minas Gerais, Belo Horizonte, Brazil

**Keywords:** *Leishmania infantum*, Visceral Leishmaniasis, Laser Direct-Write, Lateral Flow Device, immunochromatographic test

## Abstract

Visceral Leishmaniasis (VL) causes high morbidity and mortality in low-to-middle-income countries worldwide. In this study, we used Laser Direct-Write (LDW) technology to develop a new Lateral Flow Device (LFD) with double-channel geometry on a low-cost paper platform as a rapid and accurate serodiagnostic assay for human VL. This Duplex VL-LFD was based on a laser-patterned microfluidic device using two recombinant *Leishmania* proteins, β-tubulin and LiHyp1, as novel diagnostic antigens. The VL-LFD assay was tested with blood/serum samples from patients diagnosed with VL, Tegumentary Leishmaniasis, Leishmaniasis of unknown identity, other parasitic diseases with similar clinical symptoms, i.e. Leprosy Disease and Chagas Disease, and blood from healthy donors, and compared in parallel with commercial rK39 IT-LEISH^®^ Kit. Clinical diagnosis and real-time Polymerase Chain Reaction assay were used as reference standards. VL-LFD Sensitivity (S ± 95% Confidence Intervals (CI)) of 90.9 (78.9-100) and Specificity (Sp ± 95% CI) of 98.7 (96.1-100) outperformed the IT-LEISH^®^ Kit [*S* = 77.3 (59.8-94.8), Sp = 94.7 (89.6-99.8)]. This is the first study reporting successful development of an LFD assay using the LDW technology and the VL-LFD warrants comparative testing in larger patient cohorts and in areas with endemic VL in order to improve diagnosis and disease management.

## Introduction

Leishmaniasis is a disease caused by protozoan parasites of the genus *Leishmania* and is considered endemic in 98 countries, with the highest burden of disease in India, Brazil and other low-to-middle-income countries (LMIC). The most severe form of the disease is Visceral Leishmaniasis (VL), known also as Kala-Azar, which is caused by *L. chagasi/L. infantum* in the Americas and *L. donovani* and *L. infantum* in Afro-Eurasia [1]. Each year, 200,000–400,000 new cases of VL are reported, with as many as 50,000 deaths. Without treatment, the mortality rate for VL is believed to approach 100%. Even with appropriate treatment, mortality rates are between 5% and 10% and patients can relapse within a year after infection [2].

Rapid and precise diagnosis of human VL is needed to treat an otherwise fatal disease. Currently, VL is diagnosed by combining clinical manifestations with molecular, parasitological or serological tests. However, molecular assays require specialized equipment and personnel and parasitological diagnosis is affected by variability in detection sensitivity and by the expertise of the pathologist. Conventional serological tests, such as indirect immunofluorescence (RIFI) and ELISA [3], use whole pathogen or soluble extracts as antigens, but these antigen mixtures lead to cross-reactivity with other diseases and decrease specificity, and therefore should be interpreted with caution [4].

In recent years, the most widely used antibody-detecting diagnostic tests commercially available are immunochromatographic tests (ICTs), also called lateral flow tests, “dipstick” or tape tests, e.g. Kalazar Detect® and IT-LEISH® [5], which are all based on using rK39 derived from *L. chagasi/L. infantum* as the antigen. These diagnostic methods are used increasingly in endemic countries for detecting patients with VL, but the problems of inaccuracy, sub-optimal sensitivity and specificity persist.

The development of novel, effective and affordable assays for diagnosing VL rapidly and for guiding treatment is a key requirement for VL eradication. We have previously shown the usefulness and versatility of a Laser Direct-Write (LDW) approach to manufacture diagnostic devices in porous materials, such as cellulose [6, 7] and nitrocellulose [8], as well as in creating 3D structures in such membranes [9]. We have also shown the capabilities of the LDW technique in developing enhanced Lateral Flow Devices (LFDs) for multiplexing [10] and high sensitivity [11]. LDW has intrinsic advantages over alternative approaches for patterning membranes, e.g. photolithography [12], wax printing [13], inkjet printing [14], laser cutting [15], plasma treatment [16] and flexographic printing [17]. LDW is a non-lithographic approach with high flexibility that makes it ideal for rapid prototyping, and with small upfront equipment costs and no special laboratory and material requirements, it has the potential to be up-scaled for mass-production of paper-based Point-Of-Care devices.

In this study, we used the LDW method to develop a new laser-patterned microfluidic device on a low-cost paper platform with double-channel geometry as a rapid serodiagnostic assay for human VL and compared it with a commercial rK39-based “dipstick” assay. Notably, the new VL-LFD contains two *Leishmania* proteins in their recombinant forms, β-tubulin and LiHyp1 [18, 19], as novel diagnostics antigens.

## Materials and methods

### Serum samples

Patients with Visceral Leishmaniasis (VL, *n* = 24), Tegumentary Leishmaniasis (TL, *n* = 27), Leishmaniasis of unknown identity (L, *n* = 3) and Unknown Infection (UI, *n* = 49) were diagnosed by clinical evaluation, compatible clinical symptoms, conventional ELISA and/or detection of *L. infantum* (strain MHOM/BR/1970/BH46) kinetoplastid (k)DNA in bone marrow aspirates by *Leishmania-*quantitative Real-Time-PCR (L-qRT-PCR) technique. None of the VL or TL patients had been previously treated with anti-leishmanial drugs before blood sample collection. Samples from patients with confirmed Chagas Disease (CD, *n* = 53) and Leprosy Disease (LD, *n* = 13) were also collected and tested by L-qRT-PCR to exclude diagnosis of Leishmaniasis. Blood samples obtained from healthy donors (H, *n* = 20) were used as negative controls.

### Ethics statement

This study was approved by the Human Ethics Committee from the Federal University of Minas Gerais (UFMG), Belo Horizonte, Minas Gerais, Brazil (protocol number CAAE e 32343114.9.0000.5149).

### Quantitative real-time PCR

*Leishmania*-specific (L-qRT-PCR), Chagas Disease-specific (CD-qRT-PCR) and Leprosy Disease-specific (LD-qRT-PCR) quantitative real-time polymerase chain reactions were done using pathogen-specific primers (see Supplementary Table 1).

### Cloning, expression and purification of recombinant β-tubulin and LiHyp1 proteins

The gene sequence encoding for rβ-tubulin (LbrM.33.0920) was amplified by PCR using *L. braziliensis* genomic DNA as a template and cloned into pET28a-TEV vector. The coding sequence for LiHyp1 protein (LinJ.35.1290) was amplified by PCR using *L. infantum* genomic DNA as a template and cloned into pET21a vector. rβ-tubulin and rLiHyp1 proteins were purified by Ni-IDA affinity chromatography.

### Preparation of lateral flow biosensors

LDW technology was used to produce the dual-channel VL-LFD, as described previously [6, 8, 9].

### VL serodiagnosis

1. *Duplex LFD assay.* A sandwich format was used for the VL assay implemented via the LFDs. A microtitre well (of a 96 well microplate) was filled with 15 μL of detection antibody (Immunogold conjugate goat anti-human IgG (H + L), 40 nm gold) at a concentration of 1.6 μg/mL in 0.5% (v/v) Tween 20 in PBS, 15 μL of patient serum and 15 μL of fresh whole blood with heparin (collected from healthy donors). The LFD was immersed in the conjugate antibody-serum sample solution, and the device was then left to run for ∼5 min until all of the solution was wicked through the membrane. The result was immediately documented using a standard scanner.
2. *IT-LEISH® Rapid Test analysis.* Whole blood sample analysis using the commercial IT-LEISH® Kit (Bio-Rad, catalog 710124) was done following the manufacturer's instructions.

### Statistical analysis

Sensitivity (S) and Specificity (Sp) values for the Duplex VL-LFD and the commercial kit were calculated in Microsoft Excel assuming normal probability distribution.

### Supplementary methods

This section contains full details of all methods used.

## Results

### Classification of samples by clinical diagnosis and qRT-PCR analysis

A total of 169 patients were assessed clinically and blood samples were collected for further analysis. On initial clinical diagnosis, samples were collected from patients classified with VL (*n* = 24), Tegumentary Leishmaniasis (TL, *n* = 27), Leishmaniasis of unknown identity (L, *n* = 3), other diseases with similar clinical symptoms, i.e. Leprosy Disease (LD, *n* = 13) and Chagas Disease (CD, *n* = 53, with either Chagasic Myocardiopathy (CM) or Indeterminate Form (IF) of CD) or Unknown Infection (UI, *n* = 49, comprising of patients assessed with diagnosis Not Informed (NI), Anaemia (A) or Lymphoproliferative (LP)). Samples were also obtained from healthy donors as controls (H, *n* = 20).

VL, TL, L, UI and H blood samples were tested by *Leishmania*-qRT-PCR to confirm clinical diagnosis. L-qRT-PCR is specific for Leishmaniasis but unable to distinguish between different *Leishmania* species. Therefore, for samples that were L-qRT-PCR-positive, the clinical diagnosis was critical for classifying the disease as VL or non-VL (i.e. TL), and for samples that were L-qRT-PCR-negative this excluded the possibility of *Leishmania* infection of any type. Samples initially diagnosed with VL, but which tested negative in L-qRT-PCR (i.e. L006 and L009; see Supplementary Table 2), were re-classified as Not Informed (NI, see Supplementary Table 3). Samples from patients with initial UI (i.e. L016, L021, L039, L052, L053, L068, L072 and L073) and samples from healthy volunteers (i.e. CN54, CN55, CN60 and CN63) which tested positive for L-qRT-PCR (see Supplementary Table 2) were all re-classified as L (VL or TL, see Supplementary Table 3).

Patients diagnosed clinically with LD were positive in the LD-qRT-PCR. Patients diagnosed clinically with CD were also confirmed positive by CD-qRT-PCR (see Supplementary Table 2). All of these LD and CD samples were negative in the L-qRT-PCR assay, thus confirming no Leishmaniasis.

### Development and testing of a novel LDW-LFD for rapid diagnosis of human VL

**Figure 1.**
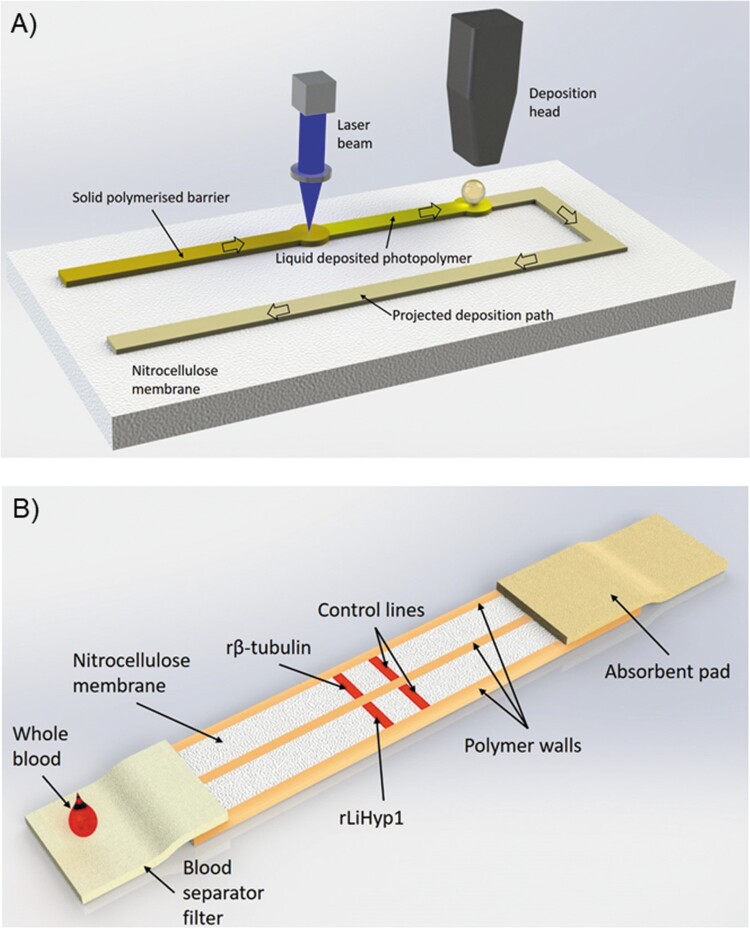
(A) LDW setup. Scheme of the laser-based direct-write setup, which shows the deposition head printing the liquid photopolymer onto the nitrocellulose membrane and its subsequent photo-polymerization by the laser beam. (B) Duplex LFD specific for human VL (VL-LFD). Schematic diagram of the proposed new LDW LFD with double-channel geometry as a rapid serodiagnostic assay for human VL. The assay is based on a laser-patterned microfluidic device on a low-cost paper platform, using recombinant β-tubulin and LiHyp1 antigens.

The LDW setup that allows implementation of this methodology is illustrated in Figure 1(A) and displayed in Video 1. The photopolymer is locally deposited using a specialized dispenser onto the nitrocellulose membrane at pre-defined locations according to the required design of the device. Following the polymer deposition step, a laser beam follows the printed design and illuminates the deposited patterns thereby inducing photo-polymerization of the polymer. Photo-polymerization turns the polymer from a liquid state to a solid state that is impermeable to liquids. Therefore, the polymerized patterns act as walls that define the boundaries of the device that confine and guide the liquid samples and reagents through the LFD. In this study, we used the LDW technique to split a standard 5 mm wide LFD into two 2.5 mm individual channels that allowed testing of two assays simultaneously without any cross-reaction between them (Figure 1). Additionally, this duplex device did not require multiple inlets or increased sample volumes to operate. Following LDW fabrication of the LFDs, we used a reagent dispensing system for the local deposition of the capture antigens onto the nitrocellulose membrane at the pre-specified test zones.

The principle of our Duplex VL-LFD assay is the “sandwich format” [20], whereby the sample Analyte (the *Leishmania*-specific antibody) binds to a Detection antibody conjugated to gold nanoparticles, and this complex is transported along the strip until it encounters the Capture antigen, i.e. rβ-tubulin or rLiHyp1, which is immobilized locally on the nitrocellulose membrane in the form of a test line. A positive control line is also included containing immobilized antibodies that are specific to the labelled detection antibody (Figure 1B).

The Duplex VL-LFD assay was validated with all VL and non-VL patient samples and its performance was compared to a commercial rapid diagnostic test (see Supplementary Table 3). S and Sp values for both assays are shown in Table 1. Out of the 22 L-qRT-PCR-positive samples from patients that were diagnosed clinically with VL infection, 20 [S = 90.9 (78.9–100)] tested positive with the Duplex VL-LFD, whereas 17 [S = 77.3 (59.8–94.8)] tested positive with the commercial kit (Table 1, Supplementary Table 3). Notably, the observation that sample L025 tested negative with both assays may suggest that the original diagnosis of VL was incorrect and may in fact be TL (see Supplementary Table 3).

**Table 1.** Selection criteria for statistics and sensitivity (S) and specificity (Sp) values for the Duplex VL-LFD and commercial kit.

Clinical diagnosis	Statistical classification	New DUPLEX VL-LFD		IT LEISH® Kit	
		% Sensitivity for VL (S, 95% CI)	% Specificity for VL (Sp, 95% CI)	% Sensitivity for VL (S, 95% CI)	% Specificity for VL (Sp, 95% CI)
VL	VL	90.9 (78.9–100)	98.7 (96.1–100)	77.3 (59.8–94.8)	94.7 (89.6–99.8)
TL	Not VL				
L	Excluded				
LD	Not VL				
CD	Not VL				
UI	Not VL				
H	Not VL				

Within the re-classified groups, samples clinically diagnosed with VL and L-qRT-PCR positive were considered “gold standard” for VL infection. Positive L-qRT-PCR reactivity of samples clinically diagnosed with Tegumentary Leishmaniasis (TL) was considered as confirmation of TL infection, therefore discarding VL (Not VL). All samples from patients diagnosed with Leishmaniasis (L) (but not specifying its type) were excluded from the statistical analysis as no confirmation for either VL or Not VL infection was available. For all Leprosy Disease (LD), Chagas Disease (CD), Unknown Infection (UI) and Healthy control (H) samples, no *Leishmania* infection was confirmed by a negative L-qRT-PCR result, and therefore all were considered as Not VL for statistical purposes. Sensitivity (S) and Specificity (Sp) values with 95% Confidence Intervals (CI) for the new Duplex VL-LFD are compared to the performance of the commercial assay tested with the same set of samples (see Supplementary Table 3).

The number of non-VL samples tested with the commercial test was restricted due to its high cost. However, all of the non-VL samples (*n* = 152) were screened with the in-house manufactured new Duplex VL-LFD. With only 1/152 false-positive samples, the new VL-LFD performed with an Sp value of 99.3 (98.0–100). For direct comparison of the new Duplex VL-LFD performance with the commercial test, however, Sp was calculated based on all non-VL samples tested with both devices only (*n* = 75). With a total of four (5%) false-positive results, the commercial test displayed an Sp value of 94.7 (89.6–99.8), which was outperformed by the new Duplex VL-LFD assay with which only one sample (1%) tested false-positive, resulting in an improved Sp value of 98.7 (96.1–100) (Table 1). The L group was excluded from statistical analyses, since no clear clinical diagnosis of VL or TL was available (Table 1).

**VIDEO 1**

https://www.dropbox.com/s/r4wfz0abzdkpzdm/My%20Movie.mp4?dl=0

**Video 1. Manufacturing and testing of a new duplex lateral flow device for human Visceral Leishmaniasis diagnosis (Duplex VL-LFD) using the Laser Direct-Write (LDW) technology.** The video highlights polymer printing, laser scanning (photo-polymerization) and antigen printing as the main steps of the manufacturing process of the new Duplex VL-LFD. A representative trial of the device with a positive VL and a negative VL sample is also shown as an example of assay performance.

**Figure 2.**
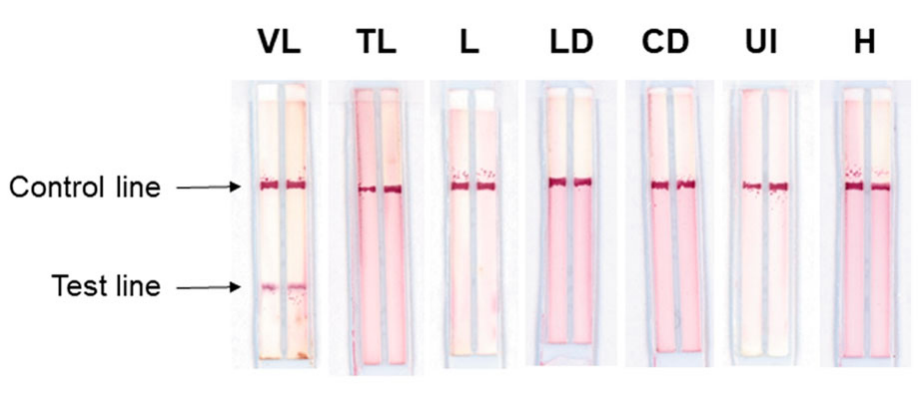
Sample testing with Duplex VL-LFD. Test strips showing a positive result for VL, and negative reactivity with Tegumentary Leishmaniasis (TL), Leishmaniasis of undetermined form (L), Leprosy Disease (LD), Chagas Disease (CD), Unknown Infection (UI), and with samples from healthy donors (H). Test results were obtained within 5 min.

Figure 2 depicts examples of the Duplex VL-LFD used for testing the positive VL samples and the TL, L, LD, CD, UI and H samples described above. In use, the Duplex VL-LFD assay always produced positive or negative reactivity with both the rβ-tubulin and rLiHyp1 antigens; thus no discordance was observed between the two antigens for all of the 189 samples tested. In all of the experiments done with both the Duplex VL-LFD and the commercial assay, the control line in both assays was displayed, thus validating the test sample data.

## Discussion

Undiagnosed VL in many LMICs can impact on management of endemic disease [5, 21]. In our study, we used the LDW approach for manufacturing paper-based rapid serodiagnostic assays to develop a highly sensitive and specific Duplex VL-LFD using two *Leishmania* antigens, β-tubulin and LiHyp1 [18, 19, 22]. The *β-tubulin* gene is expressed throughout the entire life cycle of the parasite and anti-tubulin antibodies are present in canine VL sera [19, 22]. LiHyp1 is an alkylated DNA repair protein belonging to the super-oxygenase family in *Leishmania*, and it is expressed during the amastigote (human) stage of the parasite [18, 23, 24]. Both proteins are present during the amastigote stage and were detected by *Leishmania* immunoproteomics [22].

The LDW technique allows the manufacture of double-channel LFDs with the same footprint as a standard LFD, by ensuring that the reagents needed for individual detection are kept separate in their respective flow channels. To our knowledge, our group has been the first to report the use of a dual-channel device for a true multiplexed detection [10], and this was made possible through the use of this innovative LDW step. In the absence of this unique dual-channel detection protocol, sample testing would require two separate LFDs, thus doubling the needs for reagent and sample volumes and subsequently the cost of the diagnostic testing. Using such a dual-channel device also reaffirms the validity of the results, as each sample is simultaneously and independently tested against two antigens with minimal additional cost.

The VL-LFD was used to test blood samples from 169 patients presenting with a variety of diseases and from 20 healthy donors. Our Duplex VL-LFD assay had S value of 90.9 (78.9–100) and Sp value of 98.7 (96.1–100), which was superior to the rK39 antigen-based commercial assay [S = 77.3 (59.8–94.8), Sp = 94.7 (89.6–99.8)], when both were tested in parallel with the same sample set. Performance of our VL-LFD assay can be compared also with several rK39-based ICTs in other studies and systematic reviews. Maia et al. in 2012 examined 13 studies from Brazil, India, Nepal, Tunisia, Italy and Kuwait and derived an overall S of 92% and Sp of 81% [25]. In 2014, Boelaert et al. published a Cochrane analysis of the performance of rK39 tests in several countries from the Indian subcontinent, eastern Africa, Latin America and the Mediterranean region, and reported an overall S of 91.9% (84.8–96.5) and Sp of 92.4% (85.6–96.8) [26]. More recently, rK39-ICTs were used in India to specifically detect IgG1 antibody, which improved monitoring of treatment outcomes in VL; the assays had S of ∼95–100%, but no measure of Sp was provided [27].

In general, the performance of our Duplex VL-LFD was comparable or superior to the many different rK39 ICTs used specifically in Brazilian studies (Table 2). The overall S values for these different rK39 ICTs was highly variable, ranging from 46.6 [4] to 96.0 [28], suggesting that several of these tests are inadequate for VL diagnosis in Brazil [29]. Indeed, Cunningham et al. examined the performance of different rK39 kits worldwide and found that all test brands performed well in the Indian subcontinent (S range, 92.8–100.0%; Sp range, 96.0–100.0%), but the S range was lower in Brazil and East Africa (61.5–92.0% and 36.8–87.2%, respectively), although Sp values were consistently >93% in Brazil (Table 2) and 91–98% in East Africa [29]. The S values were particularly low in the two studies done in Belo Horizonte (the same city where our study was done), using the Kalazar Detect^TM^ assay (S = 46.6–72.4%) [4, 30] but higher in the study on samples from the wider state of Minas Gerais (S = 89%) [31], although even this was still lower than the sensitivity of our Duplex VL-LFD (Table 2). These lower S values with the Kalazar Detect^TM^ assay were observed in patients infected with HIV [4, 30], which limits use of rK39 ICTs for these patients. VL diagnosis is not only hampered by HIV co-infections [32] but also by antibodies from patients with other parasitic diseases, such as Chagas Disease and Leprosy Disease [33–36] that can cross-react with high frequency, leading to a high number of false-positive results. Treatment of false-positive patients with unnecessary or inappropriate drugs could lead also to unwarranted side effects.

**Table 2.** Sensitivity (S) and Specificity (Sp) values for the Duplex VL-LFD compared with the commercial assay and with values from other published studies using rK39-based ICTs for diagnosis of human VL in Brazil.

Assay	% Sensitivity (S, 95% CI)	% Specificity (Sp, 95% CI)	Year of study	Reference
DUPLEX VL-LFD*	90.9 (78.9–100)	98.7 (96.1–100)	2019	This study
IT-LEISH®*	77.3 (59.8–94.8)	94.7 (89.6–99.8)	2019	This study
OnSite™ *Leishmania* IgG/IgM Combo	91.2 (84.5–95.1)	94.5 (86.7–97.9)	2018	[37]
DiaMed-IT LEISH®	90.0–96.0	93.0–100.0	2015	[28]
Kalazar Detect™*	72.4 (64.6–79)	99.6 (97.6–99.9)	2013	[30]
Kalazar Detect™*	46.6 (30.7–62.6)	97.1 (90.0–99.6)	2013	[4]
CrystalKA	61.5 (55.2–67.4)	98.4 (95.9–99.4)	2012	[29]
DiaMed-IT LEISH®	92.0 (87.8–94.8)	95.6 (92.2–97.5)		
Kalazar Detect™	84.7 (79.7–88.7)	96.8 (93.9–98.4)		
Signal–KA	79.2 (73.7–83.8)	98.8 (96.6–99.6)		
Kalazar Detect™ IT-LEISH®	88.1 (83.0–92.3) 93.3 (89.0–96.4)	90.6 (82.3–96.0) 96.5 (90.0–99.3)	2012	[38]
IT-LEISH®	93 (89.2–96.4)	97.0 (92.0–99.1)	2011	[39]
rK39 (TRAld)*	88.9	96.0	2009	[31]
DiaMed IT-LEISH®	93.0	97.0	2008	[40]

*These studies were done specifically in the city of Belo Horizonte in Minas Gerais State. Values for S and Sp for studies done in countries other than Brazil with endemic VL and also prior to 2008 are available in the systematic reviews from Maia et al. [25] and Boelaert et al. [26].

The performance of the IT-LEISH kit with the same sets of serum samples in our pilot study in a controlled laboratory environment showed significantly lower sensitivity (77%) than our Duplex VL-LFD assay (91%) (Table 1). However, in other studies, the sensitivity of the IT-LEISH® kit was ∼93% [38, 39], which was higher than IT-LEISH® in our study (77%), but not significantly different to the value obtained for our Duplex VL-LFD (91%), when considering confidence intervals (Table 2). A possible explanation for these differences in sensitivity values comes from the multi-centre study organized by the Special Program of Research and Training in Tropical Diseases (TDR), which reported considerable discrepancies in the performance of rapid tests in different endemic areas in Brazil (WHO/TDR 2011) [38]. This TDR study advised that it was crucial to perform regional validation before the purchase and introduction of any non-validated test [38]. Thus a larger, follow-on field study would compare the sensitivity and specificity of our Duplex VL-LFD alongside the IT-LEISH® and/or other kits within the same endemic areas in Brazil.

Our pilot study has several limitations. The Duplex VL-LFD could be a promising replacement assay for rK39-based ICTs used in the endemic area from which samples were collected in this pilot study. However, future parallel studies are needed in other endemic areas in Brazil and with a much larger number of human blood/serum samples, including from VL-HIV co-infected patients. In addition, the assay should be tested in-the-field with blood obtained by pin-pricking. The sample size of true positive VL patients (*n* = 22) in our study is similar to that reported by Cota et al. [4] in the VL-HIV co-infection study, but lower than that for many of the studies in Brazil shown in Table 2 (sample sizes of between 170 and 255 true positives) [26]. Information should be obtained also for storage temperature stability, which would require a long-term study of the Duplex VL-LFD kit alongside the IT-LEISH® kit (e.g. testing with a defined serum panel at intervals during ≥1 year storage at different temperatures). In addition, storage stability also depends on the packaging provided by commercial manufacturing, in the longer term.

The low-cost platform for VL-LFD assay development could be attractive for LMICs with remote populations suffering from endemic VL and who have poor access to health facilities, by providing a competitive cost advantage over other diagnostic ICTs commercially available. However, since the VL-LFD is at an early stage of product development, estimations of manufacturing costs would be speculative. Nevertheless, since the footprint of our Duplex VL-LFD is the same as a standard LFD, an initial sampling of a bigger patient cohort in-the-field with our novel dual-channel technology would enable us to test/validate simultaneously both antigens, without doubling the needs for reagents and blood sample volumes required for two separate LFDs. Furthermore, a larger, follow-on field study could demonstrate that both antigens behave identically with every single patient sample tested, as observed in our pilot study. In this case, removing one of the antigens could be considered, which would halve the manufacturing process, if production of the recombinant protein(s) was a major cost. Furthermore, the amount of recombinant antigen required per VL-LFD would be even less compared to a commercial single-antigen test, since the flow channel in our VL-LFD is narrower (half the width) than that of standard LFDs. In Brazil, the IT-LEISH® kit costs approximately 7.7. USD to purchase for testing one sample, but the cost of manufacture of this particular kit is not publically available information. However, manufacturing costs for a standard LFD, including packaging, is estimated at approximately 0.35 USD, and the use of laser-patterning in our process would add little extra to these costs. Thus there are probably no significant differences in manufacturing costs between our VL-LFD and standard LFDs, but a reduced purchase price would make our VL-LFD assay attractive to LMICs.

A more general limitation of rapid “dipstick” and other serological diagnostic assays for VL is that a sizeable proportion of residents of endemic areas may have antibodies present even after cure, which would lead to false-positive results. In addition, it is important to identify asymptomatic carriage of *Leishmania* parasites, since this presents a potential reservoir for transmission. However, diagnosis of the asymptomatic condition is challenging for all serological ICTs and other tests [41]. Thus our new VL-LFD should be tested further with a larger cohort of samples, including those from cured patients and asymptomatic individuals.

In summary, we have developed a new rapid serodiagnostic device for human VL using the LDW technology on a low-cost paper platform, with a double-channel geometry containing the novel recombinant protein antigens, β-tubulin and LyHyp1. This is the first study that reports successful development of an LFD assay using this technology. In direct comparison under laboratory conditions, this Duplex VL-LFD was more sensitive and specific than one currently available commercial VL diagnostic assay. This new VL-LFD warrants comparative testing in larger patient cohorts and in areas with endemic VL in order to improve diagnosis and disease management.

## Supplementary Material

Supplemental Material

## References

[1] Pace D. Leishmaniasis. J Infect. 2014;69(Suppl. 1):S10–S18. doi: 10.1016/j.jinf.2014.07.016 25238669

[2] Chappuis F, Sundar S, Hailu A, et al. Visceral leishmaniasis: what are the needs for diagnosis, treatment and control? Nat Rev Microbiol. 2007;5(11):873–882. doi: 10.1038/nrmicro1748 17938629

[3] Elmahallawy EK, Sampedro Martinez A, Rodriguez-Granger J, et al. Diagnosis of leishmaniasis. J Infect Dev Ctries. 2014;8(8):961–972. doi: 10.3855/jidc.4310 25116660

[4] Cota GF, de Sousa MR, de Freitas Nogueira BM, et al. Comparison of parasitological, serological, and molecular tests for visceral leishmaniasis in HIV-infected patients: a cross-sectional delayed-type study. Am J Trop Med Hyg. 2013;89(3):570–577. doi: 10.4269/ajtmh.13-0239 23836568 PMC3771302

[5] de Paiva-Cavalcanti M, de Morais RC, Pessoa ESR, et al. Leishmaniases diagnosis: an update on the use of immunological and molecular tools. Cell Biosci. 2015;5:31. doi: 10.1186/s13578-015-0021-2 26097678 PMC4474361

[6] Sones CL, Katis IN, He PJ, et al. Laser-induced photo-polymerisation for creation of paper-based fluidic devices. Lab Chip. 2014;14(23):4567–4574. doi: 10.1039/C4LC00850B 25286149

[7] He PJ, Katis IN, Eason RW, et al. Engineering fluidic delays in paper-based devices using laser direct-writing. Lab Chip. 2015;15(20):4054–4061. doi: 10.1039/C5LC00590F 26329148

[8] He PJ, Katis IN, Eason RW, et al. Laser-based patterning for fluidic devices in nitrocellulose. Biomicrofluidics. 2015;9(2):026503. doi: 10.1063/1.4919629 26015836 PMC4417019

[9] He PJ, Katis IN, Eason RW, et al. Laser direct-write for fabrication of three-dimensional paper-based devices. Lab Chip. 2016;16(17):3296–3303. doi: 10.1039/C6LC00789A 27436100

[10] He PJW, Katis IN, Eason RW, et al. Rapid multiplexed detection on lateral-flow devices using a laser direct-write technique. Biosensors (Basel). 2018;8(4). 10.3390/bios8040097 PMC6316105 30347807

[11] Katis IN, He PJW, Eason RW, et al. Improved sensitivity and limit-of-detection of lateral flow devices using spatial constrictions of the flow-path. Biosens Bioelectron. 2018;113:95–100. doi: 10.1016/j.bios.2018.05.001 29738945

[12] Martinez AW, Phillips ST, Wiley BJ, et al. FLASH: a rapid method for prototyping paper-based microfluidic devices. Lab Chip. 2008;8(12):2146–2150. doi: 10.1039/b811135a 19023478 PMC3065062

[13] Carrilho E, Martinez AW, Whitesides GM. Understanding wax printing: a simple micropatterning process for paper-based microfluidics. Anal Chem. 2009;81(16):7091–7095. doi: 10.1021/ac901071p 20337388

[14] Li X, Tian J, Garnier G, et al. Fabrication of paper-based microfluidic sensors by printing. Colloids Surf B Biointerfaces. 2010;76(2):564–570. doi: 10.1016/j.colsurfb.2009.12.023 20097546

[15] Fu E, Kauffman P, Lutz B, et al. Chemical signal amplification in two-dimensional paper networks. Sens Actuators B Chem. 2010;149(1):325–328. doi: 10.1016/j.snb.2010.06.024 20706615 PMC2917776

[16] Li X, Tian J, Nguyen T, et al. Paper-based microfluidic devices by plasma treatment. Anal Chem. 2008;80(23):9131–9134. doi: 10.1021/ac801729t 19551982

[17] Olkkonen J, Lehtinen K, Erho T. Flexographically printed fluidic structures in paper. Anal Chem. 2010;82(24):10246–10250. doi: 10.1021/ac1027066 21090744

[18] Martins VT, Chavez-Fumagalli MA, Costa LE, et al. Antigenicity and protective efficacy of a *Leishmania* amastigote-specific protein, member of the super-oxygenase family, against visceral leishmaniasis. PLoS Negl Trop Dis. 2013;7(3):e2148. doi: 10.1371/journal.pntd.0002148 23573301 PMC3610918

[19] Pateraki E, Portocala R, Labrousse H, et al. Antiactin and antitubulin antibodies in canine visceral leishmaniasis. Infect Immun. 1983;42(2):496–500. 6642639 10.1128/iai.42.2.496-500.1983 PMC264456

[20] Koczula KM, Gallotta A. Lateral flow assays. Essays Biochem. 2016;60(1):111–120. doi: 10.1042/EBC20150012 27365041 PMC4986465

[21] Machado de Assis TS, Azeredo-da-Silva AL, Werneck GL, et al. Cost-effectiveness analysis of diagnostic tests for human visceral leishmaniasis in Brazil. Trans R Soc Trop Med Hyg. 2016;110(8):464–471. doi: 10.1093/trstmh/trw050 27618920

[22] Coelho VT, Oliveira JS, Valadares DG, et al. Identification of proteins in promastigote and amastigote-like *Leishmania* using an immunoproteomic approach. PLoS Negl Trop Dis. 2012;6(1):e1430. doi: 10.1371/journal.pntd.0001430 22272364 PMC3260309

[23] Akhoundi M, Kuhls K, Cannet A, et al. A historical overview of the classification, evolution, and dispersion of *Leishmania* parasites and sandflies. PLoS Negl Trop Dis. 2016;10(3):e0004349. doi: 10.1371/journal.pntd.0004349 26937644 PMC4777430

[24] Teixeira DE, Benchimol M, Rodrigues JC, et al. The cell biology of *Leishmania*: how to teach using animations. PLoS Pathog. 2013;9(10):e1003594. doi: 10.1371/journal.ppat.1003594 24130476 PMC3795027

[25] Maia Z, Lirio M, Mistro S, et al. Comparative study of rK39 *Leishmania* antigen for serodiagnosis of visceral leishmaniasis: systematic review with meta-analysis. PLoS Negl Trop Dis. 2012;6(1):e1484. doi: 10.1371/journal.pntd.0001484 22303488 PMC3269412

[26] Boelaert M, Verdonck K, Menten J, et al. Rapid tests for the diagnosis of visceral leishmaniasis in patients with suspected disease. Cochrane Database Syst Rev. 2014;6:CD009135. 10.1002/14651858.CD009135.pub2 PMC4468926 24947503

[27] Mollett G, Bremer Hinckel BC, Bhattacharyya T, et al. Detection of IgG1 against rK39 improves monitoring of treatment outcome in visceral leishmaniasis. Clin Infect Dis. 2018. doi:10.1093/cid/ciy1062. PMC6743847 30541022

[28] Barbosa Junior WL, Ramos de Araujo PS, Dias de Andrade L, et al. Rapid tests and the diagnosis of visceral leishmaniasis and human immunodeficiency virus/acquired immunodeficiency syndrome coinfection. Am J Trop Med Hyg. 2015;93(5):967–969. doi: 10.4269/ajtmh.14-0798 26416105 PMC4703249

[29] Cunningham J, Hasker E, Das P, et al. A global comparative evaluation of commercial immunochromatographic rapid diagnostic tests for visceral leishmaniasis. Clin Infect Dis. 2012;55(10):1312–1319. doi: 10.1093/cid/cis716 22942208 PMC3478143

[30] Moura AS, Lopes HM, Mourao MV, et al. Performance of a rapid diagnostic test for the detection of visceral leishmaniasis in a large urban setting. Rev Soc Bras Med Trop. 2013;46(5):589–593. doi: 10.1590/0037-8682-0145-2013 24270249

[31] Romero HD, Silva Lde A, Silva-Vergara ML, et al. Comparative study of serologic tests for the diagnosis of asymptomatic visceral leishmaniasis in an endemic area. Am J Trop Med Hyg. 2009;81(1):27–33. 19556562

[32] da Silva MRB, Brandao NAA, Colovati M, et al. Performance of two immunochromatographic tests for diagnosis of visceral leishmaniasis in patients coinfected with HIV. Parasitol Res. 2018;117(2):419–427. doi: 10.1007/s00436-017-5716-3 29270768

[33] Pissinate JF, Gomes IT, Peruhype-Magalhaes V, et al. Upgrading the flow-cytometric analysis of anti-Leishmania immunoglobulins for the diagnosis of American tegumentary leishmaniasis. J Immunol Methods. 2008;336(2):193–202. doi: 10.1016/j.jim.2008.04.018 18538785

[34] Roffi J, Dedet JP, Desjeux P, et al. Detection of circulating antibodies in cutaneous leishmaniasis by enzyme-linked immunosorbent assay (ELISA). Am J Trop Med Hyg. 1980;29(2):183–189. doi: 10.4269/ajtmh.1980.29.183 6154425

[35] Kar K. Serodiagnosis of leishmaniasis. Crit Rev Microbiol. 1995;21(2):123–152. doi: 10.3109/10408419509113537 7639932

[36] Chatterjee M, Jaffe CL, Sundar S, et al. Diagnostic and prognostic potential of a competitive enzyme-linked immunosorbent assay for leishmaniasis in India. Clin Diagn Lab Immunol. 1999;6(4):550–554. 10391861 10.1128/cdli.6.4.550-554.1999 PMC95726

[37] Freire ML, Assis TSM, Avelar DM, et al. Evaluation of a new brand of immunochromatographic test for visceral leishmaniasis in Brazil made available from 2018. Rev Inst Med Trop Sao Paulo. 2018;60:e49. doi: 10.1590/s1678-9946201860049 30231169 PMC6169089

[38] Peruhype-Magalhaes V, Machado-de-Assis TS, Rabello A. Use of the Kala-Azar detect(R) and IT-LEISH(R) rapid tests for the diagnosis of visceral leishmaniasis in Brazil. Mem Inst Oswaldo Cruz. 2012;107(7):951–952. doi: 10.1590/S0074-02762012000700019 23147155

[39] de Assis TS, Braga AS, Pedras MJ, et al. Multi-centric prospective evaluation of rk39 rapid test and direct agglutination test for the diagnosis of visceral leishmaniasis in Brazil. Trans R Soc Trop Med Hyg. 2011;105(2):81–85. doi: 10.1016/j.trstmh.2010.09.004 20970152

[40] Santana Machado de Assis T, Sérgio da Costa Braga A, Junqueira Pedras M, et al. Validação do teste imunocromatográfico rápido IT-LEISH® para o diagnóstico da leishmaniose visceral humana. Epidemiol Serv Saúde. 2008;17; doi:10.5123/S1679-49742008000200004.

[41] Banuls AL, Bastien P, Pomares C, et al. Clinical pleiomorphism in human leishmaniases, with special mention of asymptomatic infection. Clin Microbiol Infect. 2011;17(10):1451–1461. doi: 10.1111/j.1469-0691.2011.03640.x 21933304

