# Supplementary material for: A rapid diagnostic test for human Visceral Leishmaniasis using novel Leishmania antigens in a Laser Direct-Write Lateral Flow Device
Source: Emerg Microbes Infect. 2019 Aug 5;8(1):1178–85. doi: 10.1080/22221751.2019.1635430 (PMC6713177; doi:10.1080/22221751.2019.1635430)
Supplement: Supplemental Material [file TEMI_A_1635430_SM3055.docx]

**Title: A rapid diagnostic test for human Visceral Leishmaniasis using novel *Leishmania* antigens in a Laser Direct-Write Lateral Flow Device**

**Authors: Maria Victoria Humbert*^1^, Lourena Emanuele Costa*^2^, Ioannis Katis*^3^, Fernanda Fonseca Ramos^2^, Amanda Sanchéz Machado^2^, Collin Sones^†3^, Eduardo Antonio Ferraz Coelho^†2,4^, Myron Christodoulides^†1#^**

**Affiliations:**

^1^ *Neisseria* Research Group, Molecular Microbiology, School of Clinical and Experimental Sciences, University of Southampton Faculty of Medicine, Southampton General Hospital, Southampton, England, SO16 6YD.

^2^ Programa de Pós-Graduação em Ciências da Saúde: Infectologia e Medicina Tropical, Faculdade de Medicina, Universidade Federal de Minas Gerais, Avenida Alfredo Balena, 190, Centro, 30.130-100, Belo Horizonte, Minas Gerais, Brazil.

^3^ Optoelectronics Research Centre, University of Southampton, Southampton SO17 1BJ, England.

^4^ Departamento de Patologia Clínica, COLTEC, Universidade Federal de Minas Gerais, Belo Horizonte, 31270-901, Minas Gerais, Brazil.

**#Author for correspondence:** Myron Christodoulides, *Neisseria* Research Group, Molecular Microbiology, School of Clinical and Experimental Sciences, University of Southampton Faculty of Medicine, Southampton General Hospital, Southampton, England, SO16 6YD. e-mail: [mc4@soton.ac.uk](mailto:mc4@soton.ac.uk). Telephone: +442380205120.

* These authors contributed equally to the experimental work; † These authors are equal principal investigators on this work.

**Keywords:** *Leishmania infantum;* Visceral Leishmaniasis; Laser Direct-Write; lateral flow device; immunochromatographic test.

**Supplementary Methods**

**Serum samples.** For this study, 169 patients attended the outpatient clinic (CTR-DIP treatment and Referral Centre in Infectious and Parasitic Diseases, Orestes Diniz, Belo Horizonte, Minas Gerais, Brazil). Patients were seen by the same doctor at all times, depending on the clinical practice. Patients with sores, Leishmaniasis, Chagas Disease and Leprosy Disease were initially attended by a single cardiologist and infectious diseases specialist and those with Leishmaniasis then referred to the chief otolaryngologist. Patients with Visceral Leishmaniasis (VL) (n = 24), Tegumentary Leishmaniasis (TL) (n = 27), Leishmaniasis of unknown identity (L) (n = 3) and Unknown Infection (UI) (n = 49) were diagnosed by means of clinical evaluation, compatible clinical symptoms, conventional ELISA and/or detection of *L. infantum* (strain MHOM/BR/1970/BH46) kinetoplastid (k)DNA in bone marrow aspirates by quantitative Real Time-PCR (L-qRT-PCR) technique. All other samples were also tested by L-qRT-PCR to exclude diagnosis of Leishmaniasis. Blood samples from patients infected with Chagas Disease (CD) (n = 53), conﬁrmed by hemoculture, Chagatest^®^ recombinant ELISA v.4.0 kit, Chagatest^®^ hemagglutination inhibition (Wiener lab, Rosario, Argentina) and CD-qRT-PCR, and patients with Leprosy Disease (LD) (n = 13), confirmed by bacillus counting, ML-Flow and LD-qRT-PCR, were also collected. None of the VL or TL patients had been previously treated with anti-leishmanial drugs before blood sample collection. Blood samples obtained from healthy donors (H) were used as negative controls (n = 20). All blood samples were collected by venipuncture of the medial vein in tubes without anticoagulant and were kept at 37°C for 15 min, centrifuged at 3,000 x g for 15 min and serum samples were separated and kept at -80°C, until use.

**Ethics Statement.** This study was approved by the Human Ethics Committee from the Federal University of Minas Gerais (UFMG), Belo Horizonte, Minas Gerais, Brazil, with a protocol number of CAAE e 32343114.9.0000.5149. All patients received an individual copy of the study policy, which was reviewed by an independent person, and all participants gave their consent form in Portuguese before the collection of their blood sample.

***Leishmania*-specific quantitative Real-Time PCR (L-qRT-PCR).** For *Leishmania*-specific quantitative Real-Time PCR reactions (L-qRT-PCR), SYBR Green^®^ system (Ludwig, Brazil) was used with primers directed to the conserved region of *Leishmania* genus mini-circle kDNA (mkDNA) (Supplementary Table 1), as described previously [1]. The PCR reaction was done in a final volume of 10 μL of a solution containing 1x SYBR Green mix; 0.4 μM of each primer and Milli-Q H_2_O to adjust to final volume. For each sample, 50 ng of DNA was used. The amplification cycles were as follows: 1 cycle of 95°C for 5 min; 40 cycles of 95°C for 30 sec, 60°C for 1 min and 72°C for 30 sec; finalized by a cycle of 72°C for 4 min. DNA extracted from promastigotes of a sample-reference *Leishmania* (*Leishmania*) *infantum* MHOM/BR/2002/LPC-RPV organism was used as a positive control. Tubes containing only sterile ultrapure water instead of the DNA samples were used as negative controls. The reactions were done on the Applied Biosystems 7500 and the data were analyzed with the Applied Biosystems 7500/7500 fast Real Time PCR software v 2.0.

**Chagas diagnosis by CD-qRT-PCR.** For Chagas Disease-specific quantitative Real-Time PCR reactions (CD-qRT-PCR), SYBR Green^®^ system (Ludwig, Brazil) was used with primers directed to the satellite region of *Trypanosoma* *cruzi* genome sequence (Supplementary Table 1) [2]. The PCR reaction was done in a final volume of 10 μL of a solution containing 1x SYBR Green mix; 0.2 μM of each primer and Milli-Q H_2_O to adjust to final volume. For each sample, 50 ng of DNA was used as a template. The cycling stages were as follows: 1 cycle of 95°C for 10 min and 40 cycles of 95°C for 15 sec and 60°C for 1 min. Patients confirmed with the disease were used as positive controls and DNA was extracted from their whole blood samples. Tubes containing only sterile ultrapure water instead of the DNA samples were used as negative controls. The reactions were done on the Applied Biosystems 7500 and the data were analyzed with the Applied Biosystems 7500/7500 fast Real Time PCR software v 2.0.

**Leprosy diagnosis by LD-qRT-PCR.** For Leprosy Disease-specific quantitative Real-Time PCR reactions (LD-qRT-PCR), SYBR Green® system (Ludwig, Brazil) was done using primers (Supplementary Table 1) directed to amplify a 129 bp sequence of the *Mycobacterium leprae* RLEP3 genome sequence, which is reported to be present at 37 copies/genome [3]. The PCR reaction was done in a final volume of 10 μL of a solution containing 1x SYBR Green mix; 0.2 μM of each primer and Milli-Q H_2_O to adjust to final volume. For each sample, 50 ng of DNA was used. The cycling stages were as follows: 1 cycle of 95°C for 5 min, 40 cycles of 95°C for 30 sec and 60°C for 1 min. Patients with the Virchowian form of the disease were used as positive controls and DNA was extracted from their whole blood samples. Tubes containing only sterile ultrapure water instead of the DNA samples were used as negative controls. The reactions were done on the Applied Biosystems 7500 and the data were analyzed with the Applied Biosystems 7500/7500 fast Real Time PCR software v 2.0.

**Cloning, expression and purification of recombinant β-tubulin and LiHyp1 proteins.** The gene sequence encoding for rβ-tubulin (LbrM.33.0920) was amplified by PCR using *L. braziliensis* genomic DNA (phenol:chloroform extracted) as a template (Supplementary Table 1). The PCR-amplified product was analyzed on an agarose gel, excised, purified, digested with NheI and SacI and finally ligated to a similarly digested pET28a-TEV vector. The recombinant plasmids were transformed into electro-competent *Escherichia coli* BL21 Arctic Express (DE3) cells (Agilent Technologies) by electroporation and sequence-checked with commercial T7 primer (Macrogen). Recombinant protein expression was induced by addition of 1 mM Isopropyl β-D-1-thiogalactopyranoside (IPTG) and further incubation for 24 h at 12°C with shaking at 200 rpm. The cells were then pelleted and lysed by sonication (applying continuous pulses of 30 s, with 15 s intervals, at 38 MHz) and centrifuged at 13,000 xg for 20 min at 4°C. rβ-tubulin protein (49.7 kDa) was soluble and purified by Ni-IDA affinity chromatography under non-denaturing conditions, using 5 mL His-Trap columns (GE Healthcare Life Science) attached to a fast protein liquid chromatography system (GE Healthcare Life Science).

The coding sequence for LiHyp1 protein (LinJ.35.1290) was amplified by PCR using *L. infantum* genomic DNA (phenol:chloroform extracted) as a template (Supplementary Table 1). The purified PCR product was cloned into the pGEM-T easy vector, sequence-verified and sub-cloned into the pET21a expression vector (Novagen). The recombinant plasmid was then transformed into competent *E. coli* BL21 (DE3) for protein expression by addition of 0.5 mM IPTG for 4 h at 37˚C. The rLiHyp1 protein (36.6 kDa) was insoluble and purified by Ni-IDA affinity chromatography under denaturing conditions with buffers containing 4M urea.

Residual endotoxin content (<10 ng of lipopolysaccharide per 1 mg of recombinant protein, measured by Quantitative Chromogenic Limulus Amebocyte Assay QCL-1000; BioWhittaker) in both recombinant protein preparations was removed by passing it through a polymyxin-agarose column (Sigma). Quality of the final purified products was assessed by Sodium Dodecyl Sulfate (SDS) -10% Polyacrylamide Gel Electrophoresis (PAGE).

**Preparation of lateral flow biosensors.** A LDW technique was used to produce the dual-channel VL-LFD, as described previously [4-6]. The schematic of this dual-channel VL-LFD with two adjoining but separate fluid-flow channels that allow the use of two different assays for the detection of the analyte within the same sample without cross-reactivity is shown in Figure 2. The schematic in Figure 1 and Video 1 show the implementation of the LDW method for the creation of the photopolymer walls that define the design of dual-channel VL-LFD. The photopolymer walls forming the boundaries of the two adjoining individual flow channels with widths of 2.5 mm were prepared by dispensing the photopolymer DeSolite 3471-3-14 (DSM Desotech, USA) with a specialized dispenser (PICO Pμlse, Nordson EFD, UK). The dispensed (liquid) material was subsequently polymerized (transforming it into a solid state) with a laser beam (405 nm continuous wave diode laser with a maximum power of 110 mW, Cobolt AB, Sweden). In order to create the solid impermeable channel walls within the nitrocellulose membrane, the photopolymer dispensing was done at a speed of 40 mm/s thus ensuring the creation of a continuous polymer line through the whole thickness of the membrane. The temperature of the dispensing head was set at 70°C to reduce the viscosity and facilitate the proper flow of the photopolymer through the dispenser. The test line (to implement the VL-assays) was subsequently produced by dispensing the antigen with a XYZ3210 Dispense Platform (Biodot, USA) dispenser (rβ-tubulin in the left channel at 0.63 mg/mL in Phosphate-buffered saline (PBS) and rLyHyp1 in the right channel at 0.5 mg/mL in PBS + 4M urea). Similarly, the control line was produced by dispensing the control antibody (anti-human IgG in 1% (w/v) Bovine Serum Albumin (BSA) in PBS) with a Biodot dispenser at a concentration of 1 mg/mL. The test lines and control lines were dispensed on the nitrocellulose membrane at a distance of 12 mm and 15 mm from the inlet of the device respectively. The reagent droplets dispensed to produce the test and control lines had a volume of 20 nL and a separation of 250 μm, and this ensured the formation of continuous test and control lines. The detection antibody was a goat anti-human IgG tagged with 40 nm gold nanoparticles with an optical density of 10 (~9 x 10^11^ gold nanoparticles per mL). The nitrocellulose membrane was then attached to a standard LFD backing card, and this was followed by attachment of the cellulose-based absorbent pad so that it overlapped the nitrocellulose membrane by 2 mm. Surplus reagents, such as an optional wash buffer, are transported toward this absorbent pad that collects unwanted/unused waste. A blood separator filter was then added at the inlet of the device overlapping the nitrocellulose membrane by 2 mm. The striped devices were left to dry at room temperature overnight and then the card was cut along the polymerized lines with a manual cutter to obtain individual LFDs.

**Visceral Leishmaniasis serodiagnosis.** *(i) Lateral Flow Device (LFD) assay***.** A sandwich format was used for the Leishmaniasis assay implemented via the LFDs. The 40 nm conjugate gold anti-human detection antibodies first bind to the Leishmaniasis analyte from the sample. The Leishmaniasis antibody then binds to the rβ-tubulin and rLyHyp1 proteins (in the test line), thus producing a colour signal at the test line only when the antibody is present in the sample. The detection antibodies also bind to the anti-human antibodies in the control line, thus producing a coloured signal regardless of the presence or absence of the Leishmaniasis antibodies in the sample. A microtiter well (of a 96 well microplate) was filled with 15 μL of the detection antibody (Immunogold conjugate goat anti-human IgG (H+L), 40 nm gold, BBI Solutions) at a concentration of 1.6 μg/mL in 0.5% (v/v) Tween 20 in PBS, 15 μL of patient serum and 15 μL of fresh whole blood with heparin (collected from healthy donors). The LFD was immersed in the conjugate antibody – serum sample solution, and the device was then left to run for several minutes (~5 min) until all of the solution was wicked through the membrane, and the result was immediately documented using a standard scanner.

*(ii) IT-LEISH^®^ Rapid Test analysis.* Whole blood sample analysis using the commercial IT-LEISH Kit (Bio-Rad, catalog 710124) was done following the manufacturer’s instructions. Briefly, the conjugate well was allowed to hydrate with 1 drop of buffer for 1 min. Then, 20 µL of whole blood sample (10 µL normal whole blood spiked with 10 µL test serum) was added and stirred gently with the conjugate. The dipstick was placed inside the well and the reaction was allowed to develop for 10 min, after which the sample/conjugate mixture was completely soaked up. The dipstick was then washed with the provided buffer until a clear background was achieved (~ 10 min). The test was considered positive when both the control line and the test line appeared red in colour. All reactions were done at room temperature.

**Statistical analyses.** Sensitivity (S) and Specificity (Sp) values for the Duplex VL-LFD and the commercial kit were calculated in Microsoft Excel assuming normal probability distribution and informed with their corresponding 95% Confidence Intervals (CI). For direct comparison of the performance of both assays, the same ‘n’ was taken into account (i.e. only those samples that have been tested with both assays), but for the Duplex VL-LFD assay in particular, with which a higher number of non-VL samples were tested, a second Sp value was also determined with the ‘higher n’ value.

| **Primer Name** | **Sequence (5’ - 3’)** | **Purpose** | **Reference** |
| --- | --- | --- | --- |
|  |  |  |  |
| L-Fw | AAGCSTCTTGCGGGGAKGGG | L-qRT-PCR | [1] |
| L-Rv | SSSWCTATWTTACACCAACCCC | L-qRT-PCR |  |
| CD-Fw | AGCTTTTGCTAGTCGGCTSA | CD-qRT-PCR | [2] |
| CD-Rv | AATTCCTCCAAGCAGCGGATA | CD-qRT-PCR |  |
| LD-Fw | TGCATGTCATGGCCTTGAGG | LD-qRT-PCR | [3] |
| LD-Rv | CACCGATACCAGCGGCAGAA | LD-qRT-PCR |  |
| βT-Fw | GCTAGCATGCGTGAGATCGTTTCCTG | β-tubulin cloning | This study |
| βT-Rv | GAGCTCCTAGTAGGCCTCCTCCTC | β-tubulin cloning |  |
| L1-Fw | GAAGGATCCAGCATGTCTATCGTGTCGAG | LiHyp1 cloning | This study |
| L1-Rv | GGAAAGCTTCGCTTGCGGCGTCACGTGAGC | LiHyp1 cloning |  |
|  |  |  |  |

**Supplementary Table 1.** **List of primers used in this study.**

The underlined sequences correspond to the restriction sites for the NheI (GCTAGC), SacI (GAGCTC), BamHI (GGATCC) and HindIII (AAGCTT) restriction enzymes. Standard abreviations are used to represent ambiguity, as follows: S (G or C), K (G or T) and W (A or T). L: Leishmaniasis; CD: Chagas Disease; LD: Leprosy Disease.

**Supplementary Table 2. Classification of samples by clinical diagnosis and qRT-PCR analysis.**

| **Patient** | **Sample ID** | **Clinical diagnosis** | **L-qRT-PCR** |
| --- | --- | --- | --- |
|  |  |  |  |
| 1 | L004 | VL | P |
| 2 | L006 | VL | N |
| 3 | L009 | VL | N |
| 4 | L011 | VL | P |
| 5 | L012 | VL | P |
| 6 | L013 | VL | P |
| 7 | L025 | VL | P |
| 8 | L028 | VL | P |
| 9 | L029 | VL | P |
| 10 | L030 | VL | P |
| 11 | L036 | VL | P |
| 12 | L037 | VL | P |
| 13 | L042 | VL | P |
| 14 | L069 | VL | P |
| 15 | L077 | VL | P |
| 16 | L083 | VL | P |
| 17 | LVH1 | VL | P |
| 18 | LVH3 | VL | P |
| 19 | LVH4 | VL | P |
| 20 | LVH5 | VL | P |
| 21 | LVH6 | VL | P |
| 22 | LVH9 | VL | P |
| 23 | LVH11 | VL | P |
| 24 | LVH12 | VL | P |
|  |  |  | **L-qRT-PCR** |
| 1 | L033 | TL | P |
| 2 | LTH21 | TL | P |
| 3 | LTH27 | TL | P |
| 4 | LTH28 | TL | P |
| 5 | LTH30 | TL | P |
| 6 | LTH31 | TL | P |
| 7 | LTH62 | TL | P |
| 8 | LTH67 | TL | P |
| 9 | LTH68 | TL | P |
| 10 | LTH72 | TL | P |
| 11 | LTH74 | TL | P |
| 12 | LTH79 | TL | P |
| 13 | LTH80 | TL | P |
| 14 | LTH81 | TL | P |
| 15 | LTH82 | TL | P |
| 16 | LTH83 | TL | P |
| 17 | LTH86 | TL | P |
| 18 | LTH87 | TL | P |
| 19 | LTH91 | TL | P |
| 20 | LTH143 | TL | P |
| 21 | LTH144 | TL | P |
| 22 | LTH146 | TL | P |
| 23 | LTH148 | TL | P |
| 24 | LTH149 | TL | P |
| 25 | LTH151 | TL | P |
| 26 | LTH152 | TL | P |
| 27 | LTH154 | TL | P |
|  |  |  | **L-qRT-PCR** |
| 1 | L005 | L | P |
| 2 | L026 | L | P |
| 3 | L040 | L | P |
|  |  |  | **LD-qRT-PCR** |
| 1 | H5 | LD | P |
| 2 | H7 | LD | P |
| 3 | H9 | LD | P |
| 4 | H14 | LD | P |
| 5 | H19 | LD | P |
| 6 | H24 | LD | P |
| 7 | H27 | LD | P |
| 8 | H29 | LD | P |
| 9 | H39 | LD | P |
| 10 | H40 | LD | P |
| 11 | H41 | LD | P |
| 12 | H42 | LD | P |
| 13 | H46 | LD | P |
|  |  |  | **CD-qRT-PCR** |
| 1 | C001 | CM | P |
| 2 | C002 | CM | P |
| 3 | C003 | CM | P |
| 4 | C004 | CM | P |
| 5 | C005 | CM | P |
| 6 | C006 | CM | P |
| 7 | C007 | CM | P |
| 8 | C008 | CM | P |
| 9 | C009 | CM | P |
| 10 | C010 | CM | P |
| 11 | C012 | CM | P |
| 12 | C013 | CM | P |
| 13 | C014 | CM | P |
| 14 | C015 | CM | P |
| 15 | C016 | CM | P |
| 16 | C017 | CM | P |
| 17 | C018 | CM | P |
| 18 | C020 | CM | P |
| 19 | C038 | CM | P |
| 20 | C039 | CM | P |
| 21 | C042 | CM | P |
| 22 | C064 | CM | P |
| 23 | DC1 | CM | P |
| 24 | DC2 | CM | P |
| 25 | DC3 | CM | P |
| 26 | DC4 | CM | P |
| 27 | DC5 | CM | P |
| 28 | DC6 | CM | P |
| 29 | DC7 | CM | P |
| 30 | DC8 | CM | P |
| 31 | DC9 | CM | P |
| 32 | DC10 | CM | P |
| 33 | C011 | IF | P |
| 34 | C019 | IF | P |
| 35 | C021 | IF | P |
| 36 | C022 | IF | P |
| 37 | C023 | IF | P |
| 38 | C024 | IF | P |
| 39 | C025 | IF | P |
| 40 | C026 | IF | P |
| 41 | C027 | IF | P |
| 42 | C028 | IF | P |
| 43 | C029 | IF | P |
| 44 | C030 | IF | P |
| 45 | C031 | IF | P |
| 46 | C032 | IF | P |
| 47 | C033 | IF | P |
| 48 | C035 | IF | P |
| 49 | C036 | IF | P |
| 50 | C037 | IF | P |
| 51 | C040 | IF | P |
| 52 | C041 | IF | P |
| 53 | C046 | IF | P |
|  |  |  | **L-qRT-PCR** |
| 1 | L014 | NI | N |
| 2 | L015 | NI | N |
| 3 | L016 | NI | P |
| 4 | L017 | NI | N |
| 5 | L021 | NI | P |
| 6 | L023 | NI | N |
| 7 | L024 | NI | N |
| 8 | L031 | NI | N |
| 9 | L039 | NI | P |
| 10 | L043 | NI | N |
| 11 | L044 | NI | N |
| 12 | L046 | NI | N |
| 13 | L047 | NI | N |
| 14 | L048 | NI | N |
| 15 | L051 | NI | N |
| 16 | L052 | NI | P |
| 17 | L053 | NI | P |
| 18 | L054 | NI | N |
| 19 | L055 | NI | N |
| 20 | L057 | NI | N |
| 21 | L058 | NI | N |
| 22 | L059 | NI | N |
| 23 | L060 | NI | N |
| 24 | L061 | NI | N |
| 25 | L063 | NI | N |
| 26 | L064 | NI | N |
| 27 | L065 | NI | N |
| 28 | L066 | NI | N |
| 29 | L068 | NI | P |
| 30 | L070 | NI | N |
| 31 | L071 | NI | N |
| 32 | L072 | NI | P |
| 33 | L073 | NI | P |
| 34 | L075 | NI | N |
| 35 | L076 | NI | N |
| 36 | L079 | NI | N |
| 37 | L080 | NI | N |
| 38 | L081 | NI | N |
| 39 | L082 | NI | N |
| 40 | L003 | A | N |
| 41 | L019 | A | N |
| 42 | L020 | A | N |
| 43 | L022 | A | N |
| 44 | L027 | A | N |
| 45 | L032 | A | N |
| 46 | L034 | A | N |
| 47 | L035 | A | N |
| 48 | L041 | A | N |
| 49 | L045 | LP | N |
|  |  |  | **L-qRT-PCR** |
| 1 | CN03 | H | N |
| 2 | CN04 | H | N |
| 3 | CN05 | H | N |
| 4 | CN06 | H | N |
| 5 | CN07 | H | N |
| 6 | CN12 | H | N |
| 7 | CN19 | H | N |
| 8 | CN21 | H | N |
| 9 | CN23 | H | N |
| 10 | CN30 | H | N |
| 11 | CN41 | H | N |
| 12 | CN47 | H | N |
| 13 | CN54 | H | P |
| 14 | CN55 | H | P |
| 15 | CN60 | H | P |
| 16 | CN63 | H | P |
| 17 | CN69 | H | N |
| 18 | CN74 | H | N |
| 19 | CN113 | H | N |
| 20 | CN115 | H | N |

All samples were grouped initially based on their clinical diagnosis only and *Leishmania* positive or negative infection was confirmed by L-qRT-PCR. LD and CD samples were also tested by LD-qRT-PCR and CD-qRT-PCR, respectively, to verify LD or CD infection. Initial clinical diagnosis for VL was considered mistaken when the samples tested negative for L-qRT-PCR (highlighted in red). Conversely, positive L-qRT-PCR reactivity observed with samples initially diagnosed as NI or H confirmed *Leishmania* infection (VL or TL) (highlighted in green). All highlighted samples (red and green) were then re-classified accordingly in Supplementary Table 3. P: positive; N: negative; VL: Visceral Leishmaniasis; TL: Tegumentary Leishmaniasis; L: Leishmaniasis; LD: Leprosy Disease; CD: Chagas Disease; CM: Chagasic Myocardiopathy; IF: Indeterminate Form; NI: not informed; A: Anemia; LP: Lymphoproliferative; H: healthy control; L-qRT-PCR: Real-time PCR using specific primers for Leishmaniasis; LD-qRT-PCR: Real-time PCR using specific primers for Leprosy Disease; CD-qRT-PCR: Real-time PCR using specific primers for Chagas Disease.

**Supplementary Table 3. Testing of samples with Duplex VL-LFD and commercial rK39-based kit.**

| **Patient** | **Sample ID** | **Clinical diagnosis** | **L-qRT-PCR** | **IT LEISH^®^ Kit (rK39)** | **New DUPLEX VL-LFD** | | **Observations** |
| --- | --- | --- | --- | --- | --- | --- | --- |
|  |  |  |  |  | **(rβ-tubulin)** | **(rLiHyp1)** |  |
| 1 | L004 | VL | P | P | P | P | True-positive for VL |
| 2 | L011 | VL | P | P | P | P |  |
| 3 | L012 | VL | P | P | P | P |  |
| 4 | L013 | VL | P | P | P | P |  |
| 5 | L025 | VL | P | N | N | N | False-negative with both tests |
| 6 | L028 | VL | P | P | P | P | True-positive for VL |
| 7 | L029 | VL | P | P | P | P |  |
| 8 | L030 | VL | P | N | P | P | False-negative with IT Kit^®^ |
| 9 | L036 | VL | P | N | P | P |  |
| 10 | L037 | VL | P | N | P | P |  |
| 11 | L042 | VL | P | N | P | P |  |
| 12 | L069 | VL | P | P | P | P | True-positive for VL |
| 13 | L077 | VL | P | P | P | P |  |
| 14 | L083 | VL | P | P | N | N | False-negative with new LFD |
| 15 | LVH1 | VL | P | P | P | P | True-positive for VL |
| 16 | LVH3 | VL | P | P | P | P |  |
| 17 | LVH4 | VL | P | P | P | P |  |
| 18 | LVH5 | VL | P | P | P | P |  |
| 19 | LVH6 | VL | P | P | P | P |  |
| 20 | LVH9 | VL | P | P | P | P |  |
| 21 | LVH11 | VL | P | P | P | P |  |
| 22 | LVH12 | VL | P | P | P | P |  |
|  |  |  | **L-qRT-PCR** |  |  |  |  |
| 1 | L033 | TL | P | N | N | N | True-negative for VL |
| 2 | LTH21 | TL | P | N | N | N |  |
| 3 | LTH27 | TL | P | nt | N | N |  |
| 4 | LTH28 | TL | P |  | N | N |  |
| 5 | LTH30 | TL | P | N | N | N |  |
| 6 | LTH31 | TL | P | N | N | N |  |
| 7 | LTH62 | TL | P | nt | N | N |  |
| 8 | LTH67 | TL | P |  | N | N |  |
| 9 | LTH68 | TL | P | N | N | N |  |
| 10 | LTH72 | TL | P | N | N | N |  |
| 11 | LTH74 | TL | P | nt | N | N |  |
| 12 | LTH79 | TL | P |  | N | N |  |
| 13 | LTH80 | TL | P | N | N | N |  |
| 14 | LTH81 | TL | P | nt | N | N |  |
| 15 | LTH82 | TL | P |  | N | N |  |
| 16 | LTH83 | TL | P |  | N | N |  |
| 17 | LTH86 | TL | P |  | N | N |  |
| 18 | LTH87 | TL | P |  | N | N |  |
| 19 | LTH91 | TL | P | N | N | N |  |
| 20 | LTH143 | TL | P | N | N | N |  |
| 21 | LTH144 | TL | P | N | N | N |  |
| 22 | LTH146 | TL | P | nt | N | N |  |
| 23 | LTH148 | TL | P |  | N | N |  |
| 24 | LTH149 | TL | P | N | N | N |  |
| 25 | LTH151 | TL | P | nt | N | N |  |
| 26 | LTH152 | TL | P |  | N | N |  |
| 27 | LTH154 | TL | P |  | N | N |  |
|  |  |  | **L-qRT-PCR** |  |  |  |  |
| 1 | L005 | L | P | P | N | N | VL/TL infection? |
| 2 | L026 | L | P | N | N | N |  |
| 3 | L040 | L | P | N | N | N |  |
| 4 | L016 | L* | P | N | P | P |  |
| 5 | L021 | L* | P | - | P | P |  |
| 6 | L039 | L* | P | P | P | P |  |
| 7 | L052 | L* | P | P | P | P |  |
| 8 | L053 | L* | P | P | P | P |  |
| 9 | L068 | L* | P | N | P | P |  |
| 10 | L072 | L* | P | - | N | N |  |
| 11 | L073 | L* | P | - | N | N |  |
| 12 | CN54 | L* | P | N | N | N |  |
| 13 | CN55 | L* | P | N | N | N |  |
| 14 | CN60 | L* | P | N | N | N |  |
| 15 | CN63 | L* | P | N | N | N |  |
|  |  |  | **LD-qRT-PCR** |  |  |  |  |
| 1 | H5 | LD | P | N | N | N | True-negative for VL |
| 2 | H7 | LD | P | N | N | N |  |
| 3 | H9 | LD | P | N | N | N |  |
| 4 | H14 | LD | P | N | N | N |  |
| 5 | H19 | LD | P | nt | N | N |  |
| 6 | H24 | LD | P |  | N | N |  |
| 7 | H27 | LD | P | P | N | N | False-positive with IT Kit^®^ |
| 8 | H29 | LD | P | N | N | N | True-negative for VL |
| 9 | H39 | LD | P | nt | N | N |  |
| 10 | H40 | LD | P | N | N | N |  |
| 11 | H41 | LD | P | N | N | N |  |
| 12 | H42 | LD | P | N | N | N |  |
| 13 | H46 | LD | P | N | N | N |  |
|  |  |  | **CD-qRT-PCR** |  |  |  |  |
| 1 | C001 | CM | P | nt | N | N | True-negative for VL |
| 2 | C002 | CM | P |  | N | N |  |
| 3 | C003 | CM | P |  | N | N |  |
| 4 | C004 | CM | P |  | N | N |  |
| 5 | C005 | CM | P |  | N | N |  |
| 6 | C006 | CM | P |  | N | N |  |
| 7 | C007 | CM | P |  | N | N |  |
| 8 | C008 | CM | P | N | N | N |  |
| 9 | C009 | CM | P | nt | N | N |  |
| 10 | C010 | CM | P | N | N | N |  |
| 11 | C012 | CM | P | nt | N | N |  |
| 12 | C013 | CM | P |  | N | N |  |
| 13 | C014 | CM | P |  | N | N |  |
| 14 | C015 | CM | P |  | N | N |  |
| 15 | C016 | CM | P |  | N | N |  |
| 16 | C017 | CM | P |  | N | N |  |
| 17 | C018 | CM | P |  | N | N |  |
| 18 | C020 | CM | P |  | N | N |  |
| 19 | C038 | CM | P |  | N | N |  |
| 20 | C039 | CM | P |  | N | N |  |
| 21 | C042 | CM | P |  | N | N |  |
| 22 | C064 | CM | P |  | N | N |  |
| 23 | DC1 | CM | P |  | N | N |  |
| 24 | DC2 | CM | P |  | N | N |  |
| 25 | DC3 | CM | P |  | N | N |  |
| 26 | DC4 | CM | P |  | N | N |  |
| 27 | DC5 | CM | P | N | N | N |  |
| 28 | DC6 | CM | P | nt | N | N |  |
| 29 | DC7 | CM | P |  | N | N |  |
| 30 | DC8 | CM | P |  | N | N |  |
| 31 | DC9 | CM | P |  | N | N |  |
| 32 | DC10 | CM | P |  | N | N |  |
| 33 | C011 | IF | P | N | N | N |  |
| 34 | C019 | IF | P | nt | N | N |  |
| 35 | C021 | IF | P |  | N | N |  |
| 36 | C022 | IF | P |  | N | N |  |
| 37 | C023 | IF | P |  | N | N |  |
| 38 | C024 | IF | P |  | N | N |  |
| 39 | C025 | IF | P | N | N | N |  |
| 40 | C026 | IF | P | nt | N | N |  |
| 41 | C027 | IF | P |  | N | N |  |
| 42 | C028 | IF | P |  | N | N |  |
| 43 | C029 | IF | P | N | N | N |  |
| 44 | C030 | IF | P | N | N | N |  |
| 45 | C031 | IF | P | nt | N | N |  |
| 46 | C032 | IF | P |  | N | N |  |
| 47 | C033 | IF | P | N | N | N |  |
| 48 | C035 | IF | P | nt | N | N |  |
| 49 | C036 | IF | P |  | N | N |  |
| 50 | C037 | IF | P |  | N | N |  |
| 51 | C040 | IF | P |  | N | N |  |
| 52 | C041 | IF | P |  | N | N |  |
| 53 | C046 | IF | P | N | N | N |  |
|  |  |  | **L-qRT-PCR** |  |  |  |  |
| 1 | L014 | NI | N | N | N | N | True-negative for VL |
| 2 | L015 | NI | N | - | N | N |  |
| 3 | L017 | NI | N | - | N | N |  |
| 4 | L023 | NI | N | N | N | N |  |
| 5 | L024 | NI | N | N | N | N |  |
| 6 | L031 | NI | N | N | N | N |  |
| 7 | L043 | NI | N | - | N | N |  |
| 8 | L044 | NI | N | N | N | N |  |
| 9 | L046 | NI | N | N | N | N |  |
| 10 | L047 | NI | N | N | N | N |  |
| 11 | L048 | NI | N | N | N | N |  |
| 12 | L051 | NI | N | N | N | N |  |
| 13 | L054 | NI | N | N | N | N |  |
| 14 | L055 | NI | N | N | N | N |  |
| 15 | L057 | NI | N | N | N | N |  |
| 16 | L058 | NI | N | N | N | N |  |
| 17 | L059 | NI | N | N | N | N |  |
| 18 | L060 | NI | N | N | N | N |  |
| 19 | L061 | NI | N | N | N | N |  |
| 20 | L063 | NI | N | N | N | N |  |
| 21 | L064 | NI | N | N | N | N |  |
| 22 | L065 | NI | N | N | N | N |  |
| 23 | L066 | NI | N | N | P | P | False-positive with new LFD |
| 24 | L070 | NI | N | N | N | N | True-negative for VL |
| 25 | L071 | NI | N | - | N | N |  |
| 26 | L075 | NI | N | P | N | N | False-positive with IT Kit^®^ |
| 27 | L076 | NI | N | N | N | N | True-negative for VL |
| 28 | L079 | NI | N | N | N | N |  |
| 29 | L080 | NI | N | - | N | N |  |
| 30 | L081 | NI | N | - | N | N |  |
| 31 | L082 | NI | N | N | N | N |  |
| 32 | L006 | NI* | N | N | N | N |  |
| 33 | L009 | NI* | N | N | N | N |  |
| 34 | L003 | A | N | N | N | N |  |
| 35 | L019 | A | N | N | N | N |  |
| 36 | L020 | A | N | P | N | N | False-positive with IT Kit^®^ |
| 37 | L022 | A | N | N | N | N | True-negative for VL |
| 38 | L027 | A | N | N | N | N |  |
| 39 | L032 | A | N | N | N | N |  |
| 40 | L034 | A | N | N | N | N |  |
| 41 | L035 | A | N | N | N | N |  |
| 42 | L041 | A | N | N | N | N |  |
| 43 | L045 | LP | N | P | N | N | False-positive with IT Kit^®^ |
|  |  |  | **L-qRT-PCR** |  |  |  |  |
| 1 | CN03 | H | N | nt | N | N | True-negative for VL |
| 2 | CN04 | H | N |  | N | N |  |
| 3 | CN05 | H | N |  | N | N |  |
| 4 | CN06 | H | N | N | N | N |  |
| 5 | CN07 | H | N | nt | N | N |  |
| 6 | CN12 | H | N | N | N | N |  |
| 7 | CN19 | H | N | N | N | N |  |
| 8 | CN21 | H | N | nt | N | N |  |
| 9 | CN23 | H | N | N | N | N |  |
| 10 | CN30 | H | N | N | N | N |  |
| 11 | CN41 | H | N | N | N | N |  |
| 12 | CN47 | H | N | nt | N | N |  |
| 13 | CN69 | H | N |  | N | N |  |
| 14 | CN74 | H | N | N | N | N |  |
| 15 | CN113 | H | N | N | N | N |  |
| 16 | CN115 | H | N | nt | N | N |  |

Samples were re-grouped in accordance with clinical diagnosis and L-qRT-PCR data, and tested for VL infection with both Duplex VL-LFD and commercial kit for comparison. Samples initially diagnosed with VL but which tested negative for L-qRT-PCR (*i.e.* L006 and L009; see Supplementary Table 2) were re-classified as NI (marked with *). Samples from patients with initial unknown infection (*i.e.* L016, L021, L039, L052, L053, L068, L072 and L073) and samples from healthy volunteers (*i.e.* CN54, CN55, CN60 and CN63) which tested positive for L-qRT-PCR (see Supplementary Table 2) were all re-classified as L (VL or TL, marked with *). All samples were tested repeatedly in 2-3 independent experiments. All LD and CD samples tested negative for L-qRT-PCR (data not shown). False-negatives and false-positives are highlighted in red and green, respectively. Samples where discrepancy between results was observed with the Duplex VL-LFD and the commercial kit are highlighted in yellow. P: positive; N: negative; -: not tested due to insufficient sample; nt: not tested; VL: Visceral Leishmaniasis; TL: Tegumentary Leishmaniasis; L: Leishmaniasis; LD: Leprosy Disease; CD: Chagas Disease; CM: Chagasic Myocardiopathy; IF: Indeterminate Form; NI: not informed; A: anemia; LP: Lymphoproliferative; H: healthy control; L-qRT-PCR: Real-time PCR using specific primers for Leishmaniasis; LD-qRT-PCR: Real-time PCR using specific primers for Leprosy Disease; CD-qRT-PCR: Real-time PCR using specific primers for Chagas Disease.

References

1. Pereira MR, Rocha-Silva F, Graciele-Melo C, et al. Comparison between conventional and real-time PCR assays for diagnosis of visceral leishmaniasis. BioMed research international. 2014;2014:639310.

2. Piron M, Fisa R, Casamitjana N, et al. Development of a real-time PCR assay for *Trypanosoma cruzi* detection in blood samples. Acta Trop. 2007 Sep;103(3):195-200.

3. Donoghue HD, Holton J, Spigelman M. PCR primers that can detect low levels of *Mycobacterium leprae* DNA. J Med Microbiol. 2001 Feb;50(2):177-82.

4. Sones CL, Katis IN, He PJ, et al. Laser-induced photo-polymerisation for creation of paper-based fluidic devices. Lab on a chip. 2014 Dec 07;14(23):4567-74.

5. He PJ, Katis IN, Eason RW, et al. Laser-based patterning for fluidic devices in nitrocellulose. Biomicrofluidics. 2015 Mar;9(2):026503.

6. He PJ, Katis IN, Eason RW, et al. Laser direct-write for fabrication of three-dimensional paper-based devices. Lab on a chip. 2016 Aug 16;16(17):3296-303.
